# Evaluating tolerability of resistant starch 2, alone and in combination with minimally fermented fibre for patients with irritable bowel syndrome: a pilot randomised controlled cross-over trial

**DOI:** 10.1017/jns.2022.9

**Published:** 2022-02-21

**Authors:** Daniel So, Chu K. Yao, Peter R. Gibson, Jane G. Muir

**Affiliations:** Department of Gastroenterology, Central Clinical School, Monash University and Alfred Health, Level 6, 99 Commercial Road, Melbourne, VIC 3004, Australia

**Keywords:** Fermentation, Fibre, Irritable bowel syndrome, Resistant starch, Sugarcane bagasse, RS2, resistant starch 2, IBS, irritable bowel syndrome

## Abstract

Resistant starch 2 (RS2) may offer therapeutic value to irritable bowel syndrome (IBS) patients particularly in combination with minimally fermented fibre, but tolerability data are lacking. The present study evaluated the tolerability of RS2, sugarcane bagasse and their combination in IBS patients and healthy controls. Following baseline, participants consumed the fibres in escalating doses lasting 3 d each: RS2 (10, 15 and 20 g/d); sugarcane bagasse (5, 10 and 15 g/d); and their combination (20, 25 and 30 g/d). Gastrointestinal symptoms were assessed daily. Six IBS patients and five controls were recruited. No differences in overall symptoms from baseline were found across the fibre doses (IBS, *P* = 0⋅586; controls, *P* = 0⋅687). For IBS patients, all RS2 doses led to increased bloating. One IBS patient did not tolerate the low combination dose and another the high sugarcane bagasse dose. Supplementation of RS2 ≤ 20 g/d caused mild symptoms and was generally tolerated in IBS patients even when combined with minimally fermented fibre.

## Introduction

Many dietary approaches have been proposed in the context of irritable bowel syndrome (IBS), with a common denominator being the manipulation of indigestible and/or slowly absorbed carbohydrates^(1)^. An example of such an approach is the low FODMAP diet, whereby intake of indigestible and slowly absorbed short-chain carbohydrates are restricted. These carbohydrates include oligosaccharide fibres that are rapidly fermented in the proximal colon, which can stimulate sensations of pain, distension and bloating in patients with visceral hypersensitivity by exerting distending forces upon the intestinal wall^(2)^.

The observations that FODMAPs induce symptoms in patients with IBS through their fermentation raise concerns regarding other fermentable carbohydrates in this patient group. Indeed, wheat bran has been reported to be poorly tolerated in patients^(3)^, while minimally and moderately fermentable carbohydrates appear to offer marginally beneficial effects^(3)^. However, bacterial fermentation of carbohydrates offers several putative beneficial effects, from promoting bacterial growth^(4)^, producing short-chain fatty acids (SCFA)^(5)^ and displacing protein fermentation, whose degradation generates metabolites with putatively harmful effects^(6)^. Therefore, avoiding carbohydrate fermentation entirely for symptom amelioration could negatively impact gut health in other ways.

Despite this, there is a paucity of information regarding the suitability of other types of fermentable carbohydrates for patients with IBS, namely types that are degraded at slower rates compared with FODMAPs in the colon, such as resistant starches (RS)^(7)^. This leaves clinicians with limited scientific rationale when advising patients on fibre intake. Indeed, the expert consensus opinion is that RS should be avoided in order to manage symptoms in this patient group^(8)^. However, no data exists to provide the basis for such a recommendation, as the suitability and tolerability of RS has yet to be evaluated in patients with IBS.

The use of RS is particularly attractive due to the belief that butyrate, an SCFA with potent health-promoting properties^(5)^, is selectively generated through their fermentation. A specific variety of type 2 resistant starch (RS2), Hi-Maize 1043, appears to be especially suitable for patients with IBS, having been shown to be fermented at a slow rate^(7)^. Moreover, evidence from healthy controls and animal models suggests that the fermentative activities of RS2 may be enhanced in combination with a ‘carrier’ fibre, enabling its fermentation to be more evenly spread through the colon, potentially ameliorating protein fermentation that is predominant distally^(9)^. Wheat bran has been previously used as the ‘carrier’, but this would be counterintuitive given its effects with IBS (as earlier). There are minimally fermented fibres available, such as sugarcane bagasse^(7)^, that may facilitate similar effects on the handling of RS2 within the colon.

While such physiological effects may offer therapeutic value to patients with IBS, studying such effects would go against expert opinion^(8)^, and therefore it would be unethical to proceed until tolerance was first assessed. The present pilot study aimed to assess the tolerability of RS2 and sugarcane bagasse, alone and in combination for patients with IBS, supplemented on top of habitual dietary intake, as well as effects on gastrointestinal symptoms, and dose-response effects of their supplementation. Given the lack of human data for sugarcane bagasse, the same effects were explored in a cohort of healthy controls.

## Methods

The study was conducted using a randomised, single-blinded, crossover design that consisted of three dietary interventions. English-speaking subjects with IBS, according to Rome IV criteria, and healthy controls with no gastrointestinal symptoms or suspicion of gastrointestinal disease, aged 18–65 years, were recruited from Melbourne, Australia between December 2018 and February 2019. Exclusion criteria included the presence of gastrointestinal or metabolic comorbidities such as coeliac disease and diabetes. Subjects that had, in the month preceding study commencement, used antibiotics or consumed pre- or probiotics were also excluded. Subjects were recruited regardless of habitual dietary intake. During the study, participants were not permitted to take pharmacologic agents to alter gastrointestinal symptoms or transit. The present study was conducted according to the guidelines laid down in the Declaration of Helsinki and all procedures were approved by the Monash University Human Research and Ethics Committee (Reference No. 12804). Written informed consent was obtained from all participants. The study was registered prospectively with the Australian New Zealand Clinical Trials Registry (No. ACTRN12618002042224).

Each participant completed a 3-d baseline period before being randomised, according to a computer-generated sequence, to one of three dietary interventions that differed in fibre content lasting 9 d each: RS2 derived from high-amylose starch (Hi-Maize 1043, Ingredion, Westchester, IL, USA); sugarcane bagasse (Tamu Group, Singapore) or their combination. Three escalating doses of fibre were evaluated during each dietary intervention, with each dose lasting 3 d each (mentioned later). After a minimum of 3-d washout period, the second randomised diet was followed for 9 d, with the same sequence was applied to the third diet (Supplementary Fig. S1). Participants, but not investigators, were blinded to the nature of the interventions.

Cereal mixes incorporating RS2 and sugarcane bagasse were produced through extrusion using a mixture of the fibres and digestible starches (polenta, quinoa flour and rice flour), and provided to participants to consume twice daily, for breakfast and as an afternoon snack. Based on their baseline food records (see later), personalised dietary advice was provided to participants to assist with maintaining 20–25 g/d of background fibre throughout the interventions. There were no instructions to modify the intake of FODMAPs. The daily fibre content of the provided meals (divided equally between both servings) was 10, 15 and 20 g RS during the RS2 arm; 5, 10 and 15 g fibre during the sugarcane bagasse arm; 20 g total fibre (8 g fibre, 12 g RS), 25 g total fibre (10 g fibre, 15 g RS) and 30 g total fibre (12 g fibre, 18 g RS) during the combination arm. These meals were visually matched and, aside from RS and fibre contents, were nutritionally matched across the dietary interventions.

Gastrointestinal symptoms were rated by participants daily during baseline and each intervention period using a 100 mm visual analogue scale (VAS) for overall gastrointestinal symptoms, abdominal pain, bloating, flatulence and dissatisfaction with stool consistency, where 0 mm indicated ‘no symptoms’ or ‘very happy’, and 100 mm represented ‘the worst symptoms experienced’ or ‘very unhappy’. Tolerance to each intervention was defined by participants successfully completing the doses evaluated without experiencing intolerable symptoms. The dietary intake of participants was recorded each day during the baseline period and every 3 d during each dietary intervention via food records. Intake data were analysed for energy, macronutrients fibre and RS contents via nutrition analysis software (Foodworks X7, Xyris Software Pty, Brisbane, Australia). Adherence to the interventions was assessed according to the proportion of the provided meals consumed: participants were considered to have adhered if ≥80 % of the meals were consumed according to food records.

No power calculations were computed for this pilot study. Data were analysed as intention to treat using R statistical software (version 4.0.2; R Foundation for Statistical Computing) and GraphPad Prism (version 9.1.1; GraphPad Software). The Shapiro–Wilk test was used to check for the normality of study data. Summary statistics are presented as median (range) unless otherwise specified.

Comparisons of baseline characteristics between individuals with IBS and healthy controls were made using the Mann–Whitney test. The analysis of gastrointestinal symptoms, averaged across 3 d per fibre dose, and dietary intake, averaged across the baseline and once per fibre dose, were performed using linear mixed-effects modelling fit by restricted maximum likelihood via the lme4 (version 1.1-26) package to assess the differences between each fibre dose of the intervention periods and baseline, with *P* values generated via the lmerTest (version 3.1-3) package. Each fibre dose was modelled as a fixed effect with participants modelled as a random effect. The normality of the models was assessed via the Shapiro–Wilk test. Where model residuals were non-normally distributed, data were normalised by log-transformation for analyses, but presented as non-transformed values. For gastrointestinal symptoms, multiple comparisons were conducted using the multcomp package (version 1.4-15) with no *post hoc* corrections made. Differences were considered significant where *P* ≤ 0⋅05.

Individual symptomatic responses were examined via the difference in overall gastrointestinal symptoms per fibre dose averaged over the 3-d period, from mean symptoms at baseline. Across each cohort, the median of these individual responses was taken as the overall change from baseline according to the dose of fibre evaluated.

## Results

Six individuals with IBS and five healthy controls were included in the study (Supplementary Fig. S2). One participant with IBS withdrew from the study after ceasing the first allocated dietary intervention following symptom exacerbation.

Baseline characteristics of the participants are outlined in Table 1. Abdominal pain tended to be higher (*P* = 0⋅091) in IBS participants compared with healthy controls, while ratings of dissatisfaction with stool consistency were significantly higher (*P* = 0⋅009). Differences in other symptoms were not statistically significant between the cohorts.

**Table 1.** Baseline characteristics of included participants

	IBS participants	Healthy controls	*P* value
Characteristics			
Number	6	5	
Female, *n* (%)	6 (100 %)	3 (60 %)	
Age, years	33 (27−43)	33 (27−43)	
Gastrointestinal symptoms			
Overall	18 (4−73)	5 (0−30)	0⋅178
Abdominal pain	11 (1−79)	2 (0−18)	0⋅091
Bloating	9 (0−88)	3 (0−65)	0⋅699
Flatulence	24 (0−68)	5 (0−60)	0⋅329
Dissatisfaction with stool consistency	54 (0−95)	10 (0−33)	0⋅009

Data are shown as median (range) unless indicated otherwise. Gastrointestinal symptoms and dietary intakes are analysed via the Mann–Whitney test.

Adherence to the interventions, according to the proportion of provided meals consumed, was excellent: 98 % of the meals were consumed for the RS2 intervention; 89 % consumed for the sugarcane bagasse intervention and 90 % consumed for the fibre combination. No changes in energy, carbohydrate or protein were observed in either cohort across the study, aside from fat content in the IBS cohort (Table 2). In both cohorts, consumption of fibre and RS differed across the study, with intake appearing to align with the intended interventional doses.

**Table 2.** Dietary intake (g, unless otherwise indicated) of IBS participants and healthy controls at baseline and across each intervention period

		Resistant starch 2			Sugarcane bagasse			Fibre combination			*P* value
	Baseline	Low	Mod	High	Low	Mod	High	Low	Mod	High	
IBS participants											
Energy, MJ	7⋅3 (5⋅3−8⋅4)	7⋅4 (6⋅2−8⋅3)	6⋅6 (4⋅4−8⋅4)	7⋅3 (5⋅9−7⋅8)	7⋅7 (4⋅3−8⋅8)	5⋅8 (4⋅2−9⋅1)	8⋅1 (5⋅9−9⋅9)	6⋅8 (4⋅6−7⋅9)	6⋅4 (5⋅5−8⋅1)	6⋅4 (5⋅5−8⋅9)	0⋅220
Protein	72 (60−125)	72 (55−137)	65 (51−116)	77 (33−94)	62 (38−110)	55 (23−134)	80 (62−113)	60 (42−109)	66 (46−119)	73 (26−126)	0⋅434
Fat	86 (57−138)	73 (45−91)	45 (25−71)	57 (50−75)	79 (14−112)	44 (13−76)	70 (44−119)	57 (38−69)	72 (37−98)	61 (48−82)	0⋅006
Carbohydrate	127 (93−204)	167 (145−242)	184 (148−231)	195 (180−218)	179 (167−204)	175 (162−250)	183 (130−278)	184 (133−212)	147 (128−182)	175 (131−196)	0⋅136
Fibre^a^	23 (16−36)	21 (16−28)	16 (10−32)	22 (13−30)	22 (19−34)	23 (17−46)	39 (24−42)	26 (20−36)	27 (24−36)	34 (25−41)	0⋅001
Resistant starch^a^	1 (0−2)	12 (11− 13)	16 (10−18)	22 (21−23)	3 (2−9)	3 (2−5)	3 (2−5)	15 (11−15)	16 (15−16)	19 (16−21)	<0⋅001
Healthy controls											
Energy, MJ	9⋅6 (8⋅2−11⋅5)	9⋅0 (7⋅4−11⋅4)	8⋅7 (4⋅6−13⋅8)	9⋅9 (7⋅0−11⋅9)	8⋅7 (5⋅3−9⋅1)	9⋅6 (8⋅3−10⋅5)	9⋅5 (5⋅8−11⋅1)	8⋅7 (6⋅5−10⋅2)	8⋅4 (7⋅6−11⋅1)	8⋅6 (6⋅5−10⋅4)	0⋅391
Protein	88 (78−128)	77 (75−130)	87 (57−116)	73 (57−129)	79 (67−97)	84 (74−107)	83 (67−107)	89 (59−111)	104 (77−117)	71 (56−99)	0⋅656
Fat	78 (54−100)	63 (42−85)	63 (24−97)	93 (31−100)	68 (32−71)	76 (73−86)	71 (49−88)	63 (53−78)	65 (59−101)	68 (51−101)	0⋅569
Carbohydrate	279 (216−346)	285 (253−280)	244 (153−596)	280 (191−399)	271 (11−346)	297 (215−332)	264 (161−405)	252 (178−317)	234 (230−363)	271 (194−295)	0⋅748
Fibre	31 (28−54)	24 (19−57)	20 (18−43)	20 (18−27)	28 (13−52)	30 (24−44)	33 (26−62)	32 (25−35)	32 (23−71)	30 (24−51)	0⋅033
Resistant starch	3 (1−4)	14 (13−19)	17 (16−24)	27 (21−28)	6 (2−7)	5 (3−10)	3 (2−10)	14 (13−16)	21 (13−22)	20 (20−24)	<0⋅001

MJ, megajoule.

Data are shown as median (range) and are analysed via linear mixed models. Comparisons are made across the intervention periods, including baseline, with no multiple comparisons made.

a Data log-transformed for analyses.

**Fig. 1.**
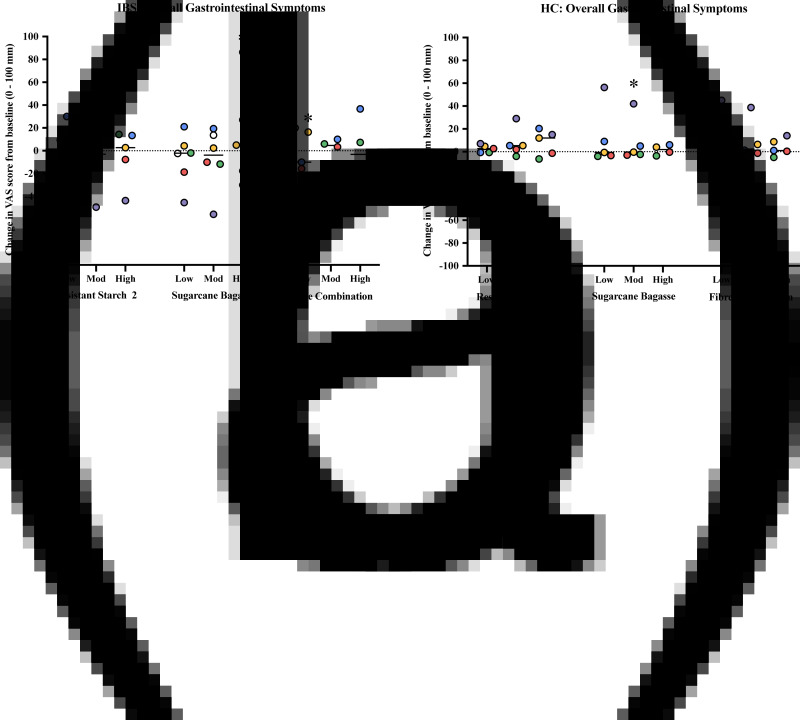
Changes in overall gastrointestinal symptoms, via visual analogue scale, from baseline during the intervention periods according to the dose of supplement given in (a) participants with IBS and (b) healthy controls. The dots represent mean changes in symptoms from baseline, per participant, according to the fibre dose; the line represents the median change in symptoms across the cohort according to the fibre dose and the asterisk indicates cessation of diets due to symptom exacerbation. Participants who ceased diets did not contribute to symptom scores and data points for subsequent doses. Individual participants are colour-coded.

In the IBS cohort, no differences in overall gastrointestinal symptoms, abdominal pain or dissatisfaction with stool consistency were found across the dietary interventions compared with the baseline period (*P* > 0⋅05) (Table 3). Bloating was higher with each dose of RS2 (*P* = 0⋅01 for all), the high dose of sugarcane bagasse (*P* = 0⋅01) as well as the moderate and high doses of the fibre combination (*P* = 0⋅02 for both). No differences were found in flatulence across the dietary interventions and baseline, aside from the high dose of the combination diet (*P* = 0⋅024). Individual symptomatic responses for the IBS cohort for overall symptoms are illustrated in Fig. 1(a). The sugarcane bagasse intervention was tolerated by five of the six IBS participants, with median differences in overall gastrointestinal symptoms of −2⋅2, −3⋅8 and 5⋅9 mm from baseline across the cohort observed during the low, moderate and high doses, respectively. One participant was unable to complete the intervention due to dose-related symptoms and withdrew from the study. Supplementation of RS2 was tolerated by all five remaining participants. Median changes in overall gastrointestinal symptoms from baseline were 9⋅3, −3⋅0 and 2⋅7 mm across the escalating doses, respectively. Four of the five IBS participants tolerated the fibre combination arm, with median differences in symptoms from baseline across the escalating doses of −10⋅0, 4⋅7 and −3⋅2 mm. One participant, who had tolerated the individual fibres across all doses, was unable to tolerate the combination at the lowest dose. One participant reported increased symptoms for all but one fibre dose and another consistently lower symptoms compared with baseline, though dose-response effects were not observed in either case.

**Table 3.** Gastrointestinal symptoms in participants with IBS, and healthy controls as assessed via visual analogue scale, at baseline and across each intervention period

		Resistant starch 2			Sugarcane bagasse			Fibre combination		
	Baseline	Low	Mod	High	Low	Mod	High	Low	Mod	High
IBS participants										
Overall^a^	18 (4−70)	25 (19−52)	25 (11−41)	30 (7−36)	19 (8−43)	21 (2−39)	35 (9–86)	20 (12–34)	30 (20−27)	27 (21–37)
Abdominal pain^a^	13 (1−57)	25 (11−31)	13 (9−42)	20 (1−41)	17 (1–36)	21 (3−42)	24 (9−87)	20 (5−33)	29 (6−42)	26 (3−57)
Bloating^a^	11 (1−75)	30 (22−76)*	35 (30−59)*	35 (20−58)*	25 (7−46)	39 (3−50)	38 (13−86)*	28 (14−42)	45 (22−55)*	38 (25−65)*
Flatulence	25 (1−64)	27 (1−77)	20 (2−64)	30 (2−72)	22 (2−73)	9 (2−52)	25 (2−54)	30 (18−44)	26 (3−59)	25 (18−78)*
Satisfaction	61 (24−70)	35 (27−62)	43 (22−53)	35 (18−68)	45 (34−74)	51 (15−87)	63 (17−99)	36 (15−78)	57 (42−66)	47 (29−64)
Healthy controls										
Overall	6 (2−15)	9 (4−20)	10 (7−42)	9 (6−11)	11 (1−69)	10 (2−55)	11 (6−55)	7 (3−58)	8 (5−52)	10 (6−27)
Abdominal pain^a^	4 (0−6)	5 (1−18)	14 (1−48)*	8 (0−11)*	10 (0−40)*	14 (1−48)*	10 (0−48)	4 (1−40)	5 (1−12)	5 (0−11)
Bloating^a^	7 (3−22)	10 (3−22)	11 (3−38)	7 (4−11)	12 (2−75)	11 (3−38)	9 (4−38)	5 (1−70)	9 (3−67)	12 (5−21)
Flatulence^a^	13 (0−24)	10 (4−43)	15 (5−50)	11 (5−14)	4 (2−50)	15 (5−50)	11 (5−50)	9 (0−77)	10 (2−82)	13 (0−84)
Satisfaction^a^	9 (0−27)	9 (1−23)	11 (3−45)	8 (0−47)	12 (0−94)	11 (0−78)	10 (2−78)	16 (2−70)	10 (0−55)	10 (0−40)

Data are shown as median (range) and are analysed via linear mixed models. Significant differences (*P* ≤ 0⋅05) across the fibre doses compared with baseline are shown via asterisk.

a Data log-transformed for analyses.

In the healthy control cohort, compared with the baseline period, abdominal pain was higher during the high dose of RS2 (*P* = 0⋅02) as well as the low and moderate doses of sugarcane bagasse (*P* = 0⋅03 for both). Compared with baseline, no other differences in gastrointestinal symptoms across the dietary interventions were found (Table 3). Median differences in overall gastrointestinal symptoms in this cohort were similar to the IBS cohort across all fibre doses (<10 mm of baseline), except for the high dose of RS2 where median symptoms were 12⋅0 mm higher. The RS2 and fibre combination interventions were tolerated by all healthy controls, while the sugarcane bagasse arm was tolerated by four of the five participants in this cohort. One participant reported non-dose-dependent increases in symptoms throughout each intervention and was unable to tolerate the sugarcane bagasse at the moderate dose (Fig. 1(b)).

## Discussion

There is currently scant literature on gastrointestinal tolerance of increasing dietary intake of RS in patients with IBS. This pilot study is the first to provide evidence that a specific type of RS (Hi-Maize 1043), a novel and minimally fermented fibre (sugarcane bagasse) and their combination were generally tolerated across escalating doses when consumed in addition to habitual intake in most patients with IBS and healthy controls. In patients with IBS, supplementation of these fibres led to increased bloating but did not impact overall gastrointestinal symptoms.

There has been much confusion over the years regarding the suitability and value of fibre therapy in IBS, partly due to the lack of fibre types evaluated and the lack of attention paid to their fermentation characteristics. Rapidly fermented fibres (fructans and galactooligosaccharides) appear to be less suitable for patients with IBS, with meta-analyses revealing their supplementation did not ameliorate symptoms and, in some cases, led to symptom exacerbation^(10)^. Indeed, dietary restriction of these fibres (alongside other short-chain carbohydrates) in the low FODMAP diet has been utilised as an effective diet therapy in IBS^(11)^. Similarly, fibre complexes containing rapidly fermented fractions, namely wheat bran, also appear to be less suitable for patients with IBS^(3)^. Conversely, psyllium, which is minimally fermented, has been shown to be broadly suitable for patients with IBS^(3)^, though its capacity to increase colonic volume^(12,13)^ may induce symptoms in certain patients^(13)^. Despite emerging data suggesting that partially hydrolysed guar gum, a moderately fermented fibre may be suitable in IBS^(14)^, the general suitability of moderate- to slowly-fermented fibres in IBS remains under-investigated. Results from this pilot study offer further evidence that these fibre types may indeed be suitable for patients with IBS.

Assessing tolerance on the basis of self-reported symptoms in patients with IBS is challenging, especially due to the fluctuating baseline in symptom severity, differences in perceptions across individuals, as well as the effects of anticipation with both placebo and nocebo effects likely to occur^(15)^. Indeed, placebo rates of 20–40 % have been reported in clinical trials conducted in IBS^(15)^, while patients may have also been prone to nocebo effects since they were aware that tolerance to the fibres was being evaluated, as observed with gluten challenges previously^(16)^. These effects were observed in this small cohort as shown by the lack of dose-dependence of many of the changes, with only one such observation occurring with sugarcane bagasse supplementation. Interestingly, the occurrence of symptom exacerbation and consistently higher symptom scores for one healthy control (Fig. 1(b)) suggest the potential presence of an underlying functional bowel disorder, given the lack of symptom response demonstrated in previous studies involving healthy individuals^(17,18)^.

The presence of sugarcane bagasse in the observed exacerbations suggests that there may be underlying mechanisms related to the fibre, independent of fermentation, that may have induced these responses, such as increases in colonic volume through its bulking actions^(9)^. Comparatively, tolerance of RS2 in the IBS cohort was good with no clear dose-response effects identified, even at the highest dose evaluated (20 g/d RS in addition to 20–25 g/d background total fibre intake), although there was evidence of increased bloating. Amongst the healthy controls, mild symptoms were observed during the highest dose of RS2, in line with previous reporting^(19)^.

The overall tolerability of RS2 and sugarcane bagasse in combination, despite the high fibre dose of 30 g/d above background intake and the greater tendency of the minimally fermented sugarcane bagasse to induce symptoms alone, was impressive. This might relate to a synergy between fermentable and minimally fermented fibres that has previously been observed in rats, pigs and humans, where fermentation of the RS2 is distributed throughout the colon rather than predominantly proximally^(9)^.

There are several limitations to this pilot study. First, individuals with IBS were recruited regardless of symptoms at baseline, which may have affected the response to the dietary interventions investigated as patients with greater initial symptoms may have experienced different effects compared with lower symptoms at baseline^(20)^. Secondly, differences in the background diet of participants, particularly involving FODMAPs and fibres, may have impacted their responses to interventions, as the dietary intake of participants, aside from instructions to maintain 20–25 g/d of background fibre intake, was uncontrolled throughout the study. Notably, the individuals with IBS had much lower intakes of FODMAPs than a typical Australian intake and were more consistent with a low FODMAP diet^(18)^ and baseline symptoms were lower compared with FODMAP-naïve patients^(18)^, which may have assisted tolerability to the fibre doses evaluated. Thirdly, the short washout between fibres evaluated may have enhanced tolerability of subsequent interventions as has been suggested with up-titration and a gradual introduction^(9)^, although these effects may have been attenuated by the randomised order of interventions. Finally, statistical analyses were limited by the small patient numbers evaluated. However, overall differences in symptoms compared with baseline across the doses evaluated remained below previous thresholds used to define clinically significant differences (10 and 20 mm)^(16,18)^, reinforcing the lack of differences found.

In addition to tolerability insights, lessons learnt from this pilot study will assist with the design of the future investigation of these fibres in patients with IBS. For example, stratification of participants based on symptom severity at baseline and greater control of dietary intake during dietary intervention periods may reduce the heterogeneity of responses and enable greater precision in the assessment of effects.

In conclusion, this pilot study showed that RS2 derived from Hi-Maize 1043 was mostly tolerated by patients with IBS when supplemented up to 20 g/d, despite mild bloating. Furthermore, the tolerance in combination with a minimally fermented fibre in sugarcane bagasse, which sometimes caused dose-dependent increases in symptoms when used alone, was surprisingly good despite the total fibre dose of 30 g on top of the background dietary intake. While no symptomatic benefits were observed, supplementation of these fibres may have beneficially modulated physiological indices not evaluated in the present study, such as promotion of SCFA generation. The results indicate that recommendations from the expert consensus regarding avoidance of RS in IBS might be displaced. A larger-scale trial investigating their physiological effects can now ethically be staged.

## Supplementary material

For supplementary material accompanying this paper visit https://doi.org/10.1017/jns.2022.9. click here to view supplementary material
