# Supplementary material for: Evaluating tolerability of resistant starch 2, alone and in combination with minimally fermented fibre for patients with irritable bowel syndrome: a pilot randomised controlled cross-over trial
Source: J Nutr Sci. 2022 Feb 21;11:e15. doi: 10.1017/jns.2022.9 (PMC8889220; doi:10.1017/jns.2022.9)
Supplement: Supplementary file 1 [file S204867902200009Xsup001.docx]

**Supplementary Figure 1.** Overview of study design of data collection processes throughout the study. As a result of the cross-over design, the second dietary intervention follows the first washout period, which is followed by the second washout period and then the third and final dietary intervention. Sequence: A, B, C, B, C, B

**Supplementary Figure 2.** CONSORT flow diagram.
